# Physicochemical characteristics, antioxidant capacity, and oxidative stress profiles of dairy and plant-based milks: a comparative multivariate analysis

**DOI:** 10.3389/fnut.2026.1948308

**Published:** 2026-10-23

**Authors:** Melek Betül Kala, Suzan Yalçın, Sıddika Songül Yalçın

**Affiliations:** 1 Institute of Health Sciences, Selcuk University, Konya, Türkiye; 2 Department of Food Hygiene and Technology, Faculty of Veterinary Medicine, Selcuk University, Konya, Türkiye; 3 Division of Social Pediatrics, Department of Pediatrics, Faculty of Medicine, Hacettepe University, Ankara, Türkiye

**Keywords:** antioxidant capacity, cow milk, oxidative balance, oxidative stress index, plant-based milk alternatives

## Abstract

**Background:**

The increasing consumption of plant-based milk (PBM) alternatives has intensified interest in their physicochemical composition and functional properties relative to dairy milk. This study comparatively evaluated the physicochemical characteristics, antioxidant capacity, and oxidative stress parameters of commercially available cow milk and selected PBMs in the Turkish market.

**Methods:**

Pasteurized and UHT cow milk, together with soy, almond, hazelnut, oat, and coconut milks, were analyzed for acidity, protein, fat, and ash contents. Antioxidant properties were assessed using total phenolic content (TPC), total antioxidant status (TAS), and catalase (CAT) activity, while oxidative balance was evaluated using total oxidant status (TOS) and oxidative stress index (OSI).

**Results:**

Cow milk had the highest protein content, followed by soy milk, whereas the other PBMs had substantially lower protein levels (median 3.10% for cow milk vs. 0.49% for PBMs overall, *p* < 0.001). PBMs had higher CAT activity (median 42.67 vs. 36.35 U/L, *p* = 0.002), whereas cow milk exhibited higher TOS (14.83 vs. 9.19 μmol H₂O₂ Eq/L, *p* < 0.001) and OSI (7.46 vs. 1.80, *p* < 0.001) PCA explained 65.3% of total variance in the first two components (PC1 45.6%, PC2 19.7%). Multivariable regression analysis identified CAT activity as the strongest independent predictor of OSI (Unstandardized *β* = −0.141, *p* < 0.001). The exploratory Global Antioxidant Quality Index (GAQI) was highest for hazelnut, soy, and oat milks.

**Conclusion:**

The findings indicate substantial variation among plant-based beverages and suggest that some PBMs may exhibit a more favorable antioxidant-oxidative balance than conventional cow milk.

## 1 Introduction

Growing awareness of the relationship between diet and the prevention of chronic diseases has increased interest in foods that offer health benefits beyond basic nutrition. In parallel, plant-based dietary patterns have gained considerable popularity due to health-related motivations, environmental sustainability concerns, ethical considerations, and evolving consumer lifestyles (1, 2). As part of this shift, consumers increasingly favor diets rich in cereals, legumes, seeds, nuts, fruits, and vegetables, leading to the emergence of dietary trends such as veganism, vegetarianism, lacto-vegetarianism, and ovo-vegetarianism (3, 4). Within this context, plant-based milk (PBM) substitutes have become one of the most widely consumed product categories, particularly among individuals with lactose intolerance, cow milk protein allergy, hypercholesterolemia, diabetes, or calorie-related dietary concerns. PBMs are also preferred during fasting periods and by consumers seeking alternatives to animal-derived foods (5). Compared to dairy milk, PBMs are often associated with lower environmental impacts, including reduced greenhouse gas emissions and water consumption (6). However, their nutritional quality and functional properties vary substantially depending on botanical origin, processing methods, and formulation strategies (7, 8). In Türkiye, official statistics indicate that drinking-milk production reached 1.64 million tonnes in 2025, representing a 3.9% increase compared with 2024, while total raw-milk production declined by 4.9% to 21.38 million tonnes in 2025. At the same time, recent consumer research conducted in Türkiye has examined purchasing behavior toward plant-based beverages and identified health, environmental, socioeconomic, and product-related factors influencing consumer choices (9–11). Taken together, these developments underscore the relevance of directly comparing commercially available dairy and plant-based beverages in the Turkish market.

PBMs are typically produced by dispersing plant-derived raw materials in water using mechanical processes such as grinding, soaking, filtration, and homogenization to form stable colloidal systems (12, 13). Structurally, PBMs consist of a continuous aqueous phase containing dispersed components, such as starch granules, protein fractions, lipid droplets, and insoluble plant matrix particles (14). They are formulated to mimic the appearance and functionality of cow milk while offering advantages related to sustainability and ethical consumption (15). A wide range of plant sources—including cereals, legumes, nuts, seeds, pseudo-cereals, and tubers—are used either individually or in blended formulations, resulting in pronounced diversity in physicochemical, nutritional, and bioactive characteristics (16–22).

Plant-based raw materials are considered functional and nutraceutical foods due to their richness in bioactive components such as minerals, essential fatty acids, non-allergenic proteins, phytosterols, isoflavones, and phenolic compounds (6, 23). Unlike cow milk, PBMs do not contain cholesterol, lactose, or certain saturated fatty acids and antigens, which may not be suitable for all consumers (17). Globally, soybeans, rice, oats, and almonds are the most commonly used raw materials in PBMs production, with soy milk remaining the dominant product due to its relatively high protein content and favorable amino acid profile (24).

Although dairy milk has traditionally been regarded as a nutritionally complete food and remains a fundamental component of human diets, increasing production costs, lactose intolerance, and sustainability-related concerns have intensified the search for suitable non-dairy alternatives. Recent evidence indicates that PBM alternatives can contribute to human nutrition by providing not only macronutrients and micronutrients, but also a variety of bioactive compounds, including polyphenols, flavonoids, anthocyanins, and other phytochemicals with potential health-promoting properties (22). However, PBMs differ substantially from dairy milk in their nutritional composition, antioxidant capacity, and oxidative stability, and comprehensive comparative studies evaluating these characteristics under standardized conditions remain limited. Previous studies have shown that PBMs generally contain lower protein, fat, and solids-not-fat contents, as well as lower acidity, compared to cow milk, reflecting differences in raw material composition and formulation strategies (5).

Therefore, the present study aimed to comprehensively compare the physicochemical properties (acidity, protein, fat, and ash content), antioxidant properties (TPC, TAS, and CAT activity), and oxidative stress parameters (TOS and OSI) of pasteurized and UHT cow milk and selected PBMs available on the Turkish market. In addition, multivariate approaches were employed to characterize relationships among compositional and oxidative parameters, identify factors associated with oxidative stress, and provide an integrated evaluation of antioxidant–oxidative profiles across different milk types.

We hypothesized that: (i) PBM alternatives would differ significantly from cow milk in terms of physicochemical composition and antioxidant–oxidative characteristics; (ii) oxidative stress would be more strongly associated with antioxidant-related variables than with milk category itself; and (iii) certain PBM alternatives would exhibit a more favorable integrated antioxidant–oxidative profile than conventional cow milk. These hypotheses were evaluated using univariate and multivariate statistical analyses together with an exploratory composite antioxidant quality index.

## 2 Materials and methods

### 2.1 Milk samples

In this study, a total of 56 commercially available milk samples offered for consumption in Türkiye were obtained. Of these samples, 16 were dairy-based milks and 40 were PBMs. The milk types included UHT cow milk, pasteurized cow milk, coconut milk, soy milk, almond milk, hazelnut milk, and oat milk. For each milk type, eight different samples were purchased from the local market. Based on the processing information provided on the product labels, all PBMs included in the study were UHT-treated, whereas the dairy milk samples comprised separate pasteurized and UHT-treated groups. The PBMs were commercially manufactured ready-to-consume products; therefore, their exact proprietary processing parameters, including specific time–temperature conditions, were not available and were not experimentally controlled in this study. In general, the products represented industrially processed beverages produced through aqueous extraction of the plant material followed by filtration and/or homogenization, with product-specific heat treatment and formulation according to the manufacturer. Processing classification was based on the information provided on the product packaging labels.

### 2.2 Determination of physicochemical properties

Protein, fat, and ash contents of dairy and PBM samples were determined according to AOAC methods (25). Titratable acidity of milk was determined according to the AOAC Official Method 947.05 (Acidity of Milk: Titrimetric Method). A 20 mL aliquot of well-mixed milk was transferred into a clean beaker, and 40 mL of boiled and cooled distilled water was added. Two milliliters of 1% phenolphthalein indicator solution (prepared in 95% ethanol) were added, and the mixture was titrated with standardized 0.1 N sodium hydroxide (NaOH) with continuous swirling, until a persistent light-pink color was observed (first endpoint). An additional single drop of 0.1 N NaOH was added to confirm the endpoint, and the total volume of NaOH consumed was recorded. Titratable acidity was calculated as mg of lactic acid per 100 mL of milk according to the following formula:


$$\text{Lactic acid}\;(\frac{\mathrm{mg}}{100\mathrm{mL}})=\frac{\left(V_{\text{NaOH}}\times N_{\text{NaOH}}\times 90\;x\;100\right)}{V_{\text{milk}}}$$

Where V_NaOH_ is the volume (mL) of standardized NaOH consumed to reach the endpoint, N_NaOH_ is the normality of the NaOH solution (Eq/L), 90 is the equivalent weight of lactic acid (g/Eq), and V_milk_ is the volume (mL) of milk sample used in the titration. The calculated titratable acidity values were subsequently converted to percentage of lactic acid (% LA) for presentation of the results. One milliliter of 0.1 N_NaOH_ is equivalent to 9.0 mg of lactic acid, in accordance with AOAC method for dairy analysis (25).

### 2.3 Determination of antioxidant capacity and oxidative stress parameters

TPC of the milk samples was determined using the Folin–Ciocalteu spectrophotometric method, with minor modifications (26, 27). Briefly, milk samples were mixed with 80% methanol at a 1:1 (v/v) ratio and centrifuged at 5000 rpm for 20 min at +4 °C. The supernatants were collected and centrifuged again at 14,900 rpm for 30 min at +4 °C. Non-clear supernatants were sequentially filtered through 0.45 μm and 0.22 μm membrane filters. Absorbance was measured at 725 nm using a spectrophotometer, with the blank used for zero adjustment. Gallic acid was used as the standard, and results were expressed as mg gallic acid equivalents (GAE)/100 mL milk. TPC values were calculated by applying the dilution factor.

For TAS and TOS analyses, 1.5 mL of each milk sample was transferred into 2 mL Eppendorf tubes and vortexed for 5 min. Samples were centrifuged at 14,900 rpm for 30 min at +4 °C, and the supernatants were collected. Non-clear samples were filtered sequentially through 0.45 μm and 0.22 μm membrane filters. TAS and TOS were measured in the clarified supernatants using commercial assay kits (Rel Assay Diagnostics, Mega Tıp San. ve Tic. Ltd. Şti., Gaziantep, Türkiye) on an automated analyzer (Mindray BS-400, China). TAS values were measured at 660 nm and expressed as mmol Trolox equivalents (TE)/L, while TOS values were measured at 530 nm and expressed as μmol H_2_O_2_ Equivalents/L. OSI was calculated using the formula of ‘OSI = ((TOS, mmol/L)*100)/(TAS, mmol/L)’ as reported by Yiğit et al. (28). Because TOS was initially expressed as μmol H_2_O_2_ Equivalents/L, TOS values were converted to mmol/L by dividing by 1,000 before OSI calculation.

The same clarified supernatants prepared for TAS and TOS analyses were also used to determine CAT enzyme activity. CAT activity was measured spectrophotometrically at 405 nm using a commercial assay kit (Elabscience Biotechnology Co., Ltd., Wuhan, China) according to the manufacturer’s instructions, and the results were expressed as U/L (29).

A composite Global Antioxidant Quality Index (GAQI) was developed for the present study as an exploratory composite index to integrate antioxidant and oxidative characteristics into a single metric for comparative purposes. Since this index is novel and has not been previously validated, it was used solely as a descriptive tool to facilitate the integrated evaluation of antioxidant-oxidative profiles among the investigated milk types. Standardized z-scores of antioxidant-related variables (TPC, CAT, and TAS) were summed, whereas standardized z-scores of oxidative parameters (TOS) were subtracted. GAQI was calculated using the following formula: [GAQI = (zTPC + zCAT + zTAS) − (zTOS)]. Higher GAQI values indicate a more favorable antioxidant–oxidative profile.

### 2.4 Statistical analysis

Statistical analyses were performed using IBM SPSS Statistics version 23 and R 4.5.1. Data distribution was assessed using histograms and the Shapiro–Wilk test. Because continuous variables were non-normally distributed, group comparisons used the Kruskal Wallis test with Bonferroni-adjusted pairwise comparisons; results are summarized primarily as medians and interquartile ranges. Spearman correlation was used for bivariate associations. For multivariate characterization, z-standardized acidity, protein, fat, ash, TPC, CAT, TAS, and TOS values were analyzed by principal component analysis (PCA) (FactoMineR/factoextra) and hierarchical clustering analysis (HCA; pheatmap). A min–max-normalized radar plot summarized TPC, CAT activity, TAS, TOS, and OSI. Multivariable linear regression modeled OSI using protein, TPC, CAT, and milk category (cow milk as reference; soy; other PBMs), with regression coefficients (β), standard errors (SEs), 95% confidence intervals (CIs), R^2^/adjusted R^2^, and the overall F statistic reported. Statistical significance was defined as *p* < 0.05; exact *p*-values are reported where available, while values below 0.001 are reported as *p* < 0.001.

## 3 Results

### 3.1 Physicochemical characteristics, antioxidant capacity and oxidative status parameters of cow milk and plant-based milk alternatives

The physicochemical properties, antioxidant capacity, and oxidative stress parameters of cow milk and selected PBMs are summarized in Table 1. Acidity values (% lactic acid) differed significantly among the milk types (*p* < 0.001), with pasteurized and UHT cow milk exhibiting the highest median values (0.16% LA) and relatively narrow interquartile ranges. All PBMs exhibited significantly lower acidity values, with oat and hazelnut milks also displaying narrow interquartile ranges, suggesting limited variability.

**Table 1:** Physicochemical characteristics, antioxidant capacity, and oxidative status parameters of cow milk and plant-based milk alternatives.

Items	Coconut milk	Soy milk	Almond milk	Hazelnut milk	Oat milk	Pasteurized cow milk	UHT cow milk	*p*-value
Acidity, % LA	0.09 (0.05)^b^	0.08 (0.12)^b^	0.09 (0.04)^b^	0.09 (0.02)^b^	0.10 (0.02)^b^	0.16 (0.01)^a^	0.16 (0.02)^a^	<0.001
Protein, %	0.28 (0.20)^b^	2.60 (1.17)^a^	0.54 (0.31)^b^	0.48 (0.12)^b^	0.41 (0.32)^b^	3.25 (0.54)^a^	3.08 (0.20)^a^	<0.001
Fat, %	1.24 (0.44)^b^	1.56 (0.22)^b^	1.16 (0.31)^b^	1.60 (0.39)^b^	1.28 (1.56)^b^	3.27 (0.73)^a^	2.10 (2.92)^b^	<0.001
Ash, %	0.45 (0.12)^bc^	0.50 (0.31)^b^	0.42 (0.17)^bc^	0.50 (0.09)^b^	0.27 (0.20)^c^	0.71 (0.02)^a^	0.69 (0.05)^a^	<0.001
TPC, mg GAE/100 mL	3.30 (3.92)^d^	14.10 (2.76)^ab^	9.43 (15.75)^bc^	7.50 (2.70)^cd^	4.00 (5.20)^cd^	14.3 (3.95)^ab^	16.5 (5.20)^a^	<0.001
CAT, U/L	43.97 (10.73)^ab^	39.45 (12.13)^abc^	34.12 (13.18)^c^	51.48 (9.64)^a^	53.91 (23.19)^a^	32.99 (12.35)^c^	36.35 (11.91)^bc^	0.001
TAS, mmol TE/L	0.14 (0.14)^c^	0.27 (0.69)^ab^	0.13 (0.10)^c^	0.77 (0.33)^a^	0.64 (0.39)^a^	0.21 (0.13)^bc^	0.21 (0.16)^bc^	<0.001
TOS, μmol H_2_O_2_ Eq/L	2.47 (4.28)^c^	11.18 (10.30)^ab^	11.70 (9.19)^ab^	9.54 (6.30)^b^	11.18 (12.57)^ab^	12.66 (8.09)^ab^	16.93 (4.53)^a^	0.001
OSI	1.82 (2.78)^b^	2.01 (7.04)^b^	9.54 (1.05)^a^	1.39 (0.67)^b^	1.76 (0.23)^b^	7.46 (3.61)^a^	7.40 (3.68)^a^	<0.001

Data are presented as median (Interquartile range, IQR); *n*: 8; abcd: values within the same row followed by different superscripts are significantly different (*p* < 0.05); LA, lactic acid; TPC, total phenolic content; GAE, gallic acid equivalent; CAT, catalase; TAS, total antioxidant status; TE, Trolox equivalent; TOS, total oxidant status; Eq, equivalent; OSI, oxidative stress index.

Protein content also varied markedly among milk types (*p* < 0.001). Median protein contents were 3.25% in pasteurized cow milk, 3.08% in UHT cow milk, and 2.60% in soy milk; these formed the highest statistical group. Coconut (0.28%), almond (0.54%), hazelnut (0.48%), and oat (0.41%) milks contained significantly lower protein levels.

Fat content differed significantly (*p* < 0.001), with pasteurized cow milk exhibiting the highest median value (3.27%). UHT cow milk had a median fat content of 2.10% and showed greater variability (IQR = 2.92) than pasteurized cow milk (IQR = 0.73). PBM median fat contents ranged from 1.16 to 1.60%.

Significant differences were also observed in ash content (*p* < 0.001). Pasteurized and UHT cow milk had the highest ash values, followed by soy and hazelnut milks, whereas oat milk exhibited the lowest mineral content. Soy milk showed the greatest variability among plant-based beverages.

TPC differed significantly among milk types (*p* < 0.001). UHT cow milk exhibited the highest median TPC (16.5 mg GAE/100 mL), while pasteurized cow milk (14.3 mg GAE/100 mL) and soy milk (14.1 mg GAE/100 mL) showed similarly high values. Coconut milk had the lowest median TPC (3.30 mg GAE/100 mL), whereas almond milk had the widest interquartile range (15.75 mg GAE/100 mL).

CAT activity also varied significantly (*p* = 0.001). Oat and hazelnut milks exhibited the highest activities, whereas pasteurized cow milk and almond milk showed the lowest values. Oat milk displayed the greatest dispersion in CAT activity.

Significant differences were observed in TAS (*p* < 0.001), with hazelnut (median 0.77 mmol TE/L) and oat milks (0.64 mmol TE/L) exhibiting the highest values, and coconut (0.14 mmol TE/L) and almond milks (0.13 mmol TE/L) the lowest. Soy milk showed a comparatively wide interquartile range (0.69 mmol TE/L).

TOS differed significantly among milk types (*p* = 0.001). UHT cow milk exhibited the highest median TOS, whereas coconut milk had the lowest median TOS value. Variability in TOS was greatest in oat milk.

OSI also differed significantly (*p* < 0.001). Almond milk and both cow milk groups exhibited the highest OSI values, while coconut, soy, hazelnut, and oat milks showed significantly lower OSI indices. Among all milk types, soy milk displayed the greatest variability in OSI.

### 3.2 Correlation patterns among physicochemical and oxidative parameters

Spearman correlation analyses were performed for all milk samples combined, separately for cow milk and plant-based milk alternatives, and for each of the seven individual milk types (Figure 1 and Supplementary Table 1). When all milk samples were analyzed together, protein content showed positive correlations with acidity (*r* = 0.50), fat (*r* = 0.42), and ash (*r* = 0.52), reflecting the compositional similarities among dairy products as shown in Figure 1. TPC was positively correlated with TOS (*r* = 0.47) and OSI (*r* = 0.43). TAS was inversely associated with OSI (*r* = −0.69), whereas TOS showed a positive correlation with OSI (*r* = 0.49), indicating that higher oxidant burden was associated with higher OSI. In cow milk samples, fewer significant correlations were observed. The strongest association was the inverse correlation between TAS and OSI (*r* = −0.75). Negative correlations were also identified between acidity and TPC (*r* = −0.62) and between fat content and TPC (*r* = −0.51), suggesting an association between compositional variation and phenolic content within dairy products.

**Figure 1:**
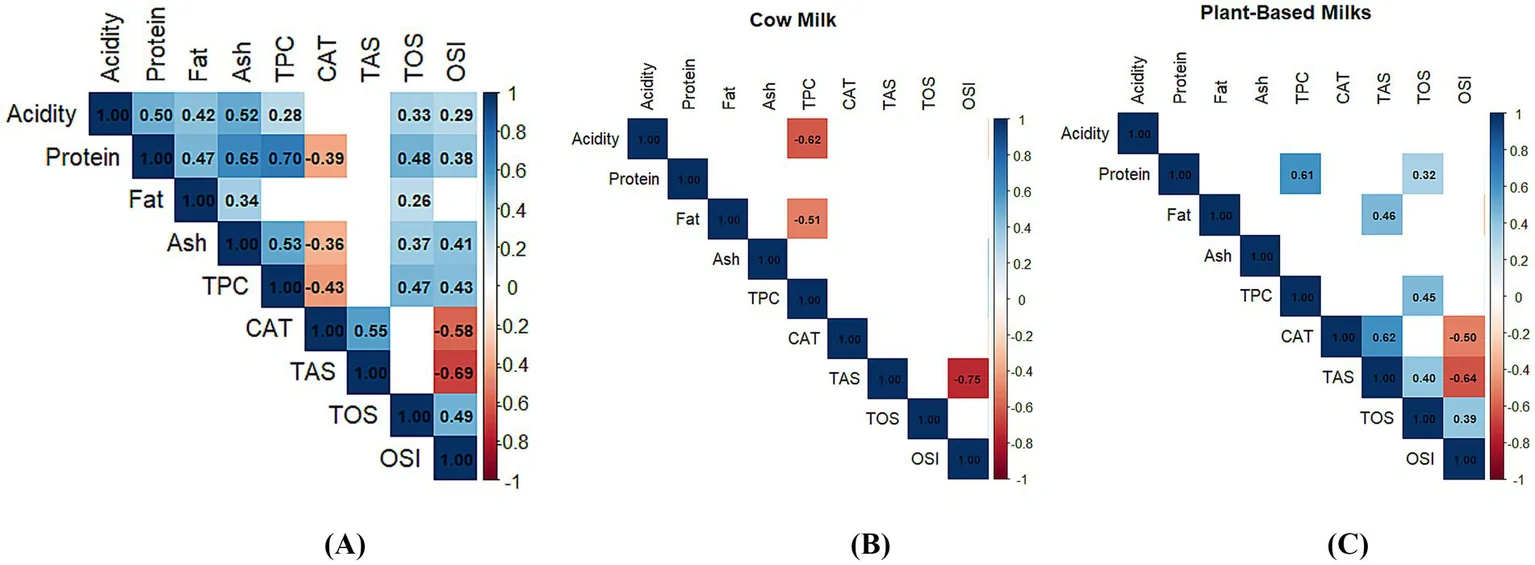
Spearman correlation matrices of physicochemical characteristics, antioxidant parameters, and oxidative stress indices for all milk samples **(A)**, cow milk samples **(B)**, and plant-based milk alternatives **(C)**. Blue cells indicate positive correlations and red cells indicate negative correlations. Only statistically significant correlations (*p* < 0.05) are displayed. TPC, total phenolic content; CAT, catalase; TAS, total antioxidant status; TOS, total oxidant status; OSI, oxidative stress index.

Among PBM alternatives, several antioxidant-related correlations were evident. CAT activity was positively associated with TAS (*r* = 0.62) and negatively associated with OSI (*r* = −0.50). TAS showed an inverse relationship with OSI (*r* = −0.64). Additionally, protein content was positively associated with TPC (*r* = 0.61), and TPC showed a moderate positive correlation with TOS (*r =* 0.45). Overall, the correlation matrices indicate that antioxidant-related parameters, particularly CAT activity and TAS, were closely associated with oxidative stress status, whereas the strength of correlations differed between dairy and PBM samples (Figure 1).

### 3.3 Multivariate characterization of milk samples

Principal component analysis explained 65.3% of the total variance within the first two principal components (PC1 = 45.6%, PC2 = 19.7%) (Figure 2). PC1 was primarily associated with protein (21.8% contribution), ash (18.7%), acidity (15.6%), and TPC (15.0%), representing compositional and nutritional characteristics. PC2 was mainly influenced by TAS (47.1% contribution), CAT activity (29.0%), and TOS (18.1%), reflecting antioxidant and oxidative properties (Supplementary Table 2).

**Figure 2:**
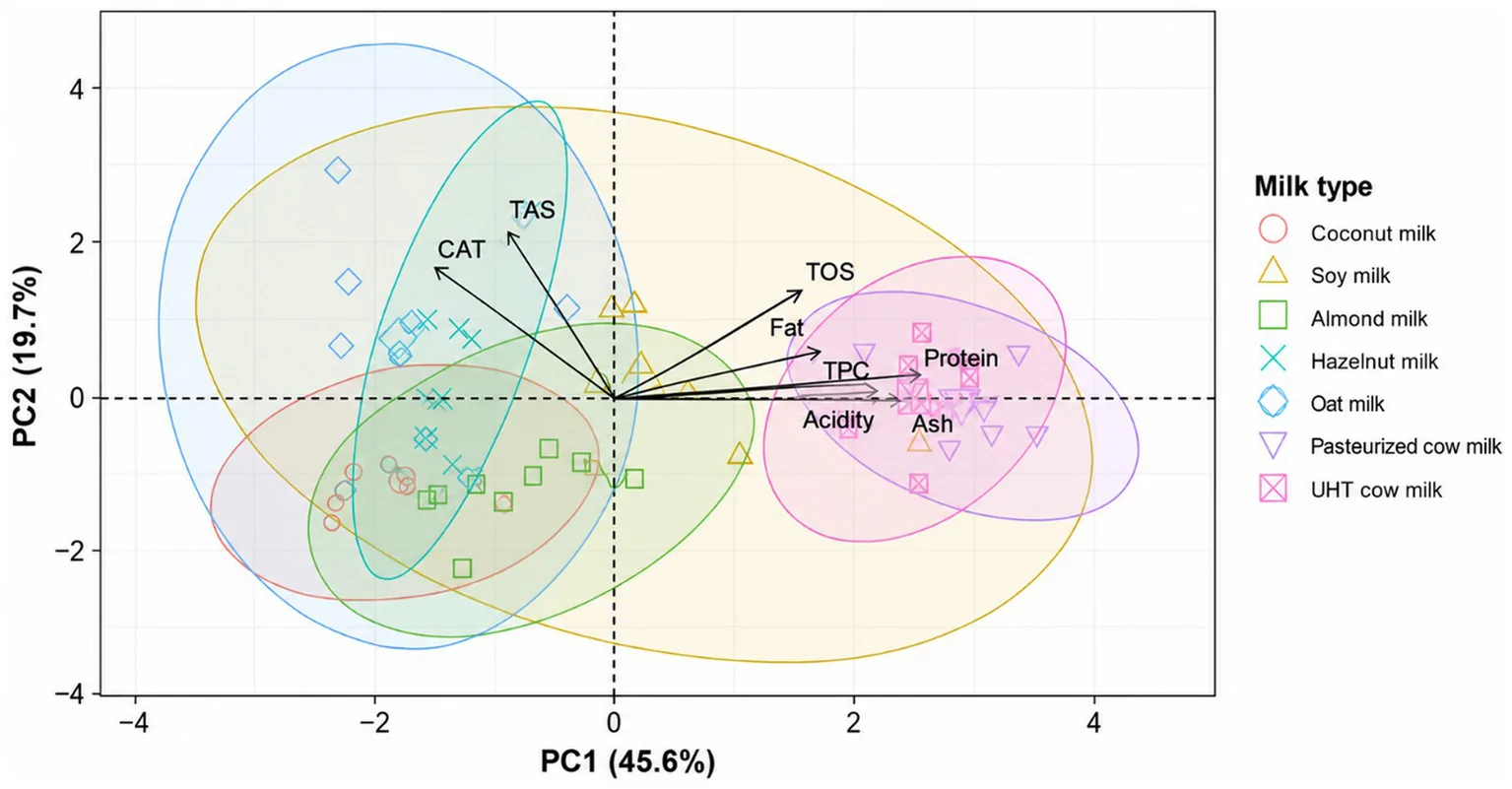
Principal component analysis (PCA) biplot of physicochemical characteristics, antioxidant capacity, and oxidative status parameters of cow milk and plant-based milk alternatives. TPC, total phenolic content; CAT, catalase; TAS, total antioxidant status, TOS, total oxidant status.

The PCA biplot demonstrated a clear separation between cow milk samples and PBM alternatives. Pasteurized and UHT cow milk clustered closely together and were associated with higher protein, ash, acidity, and TPC values. Hazelnut and oat milks were positioned along the positive PC2 axis and were characterized by higher antioxidant capacity, particularly TAS and CAT activity. Coconut and almond milks clustered separately and were associated with comparatively lower nutritional and antioxidant-related attributes, while soy milk occupied an intermediate position between dairy and other plant-based beverages.

Hierarchical clustering analysis largely confirmed the PCA findings (Figure 3, Supplementary Figure 1). Two major branches were observed, separating dairy milk samples from most plant-based alternatives. Pasteurized and UHT cow milk formed a distinct cluster characterized by higher protein, ash, acidity, and TPC values. Hazelnut and oat milks clustered together and were associated with elevated TAS and CAT activity, whereas coconut and almond milks grouped according to their lower compositional and antioxidant profiles. Soy milk formed a separate branch, reflecting its distinct nutritional composition among PBM alternatives.

**Figure 3:**
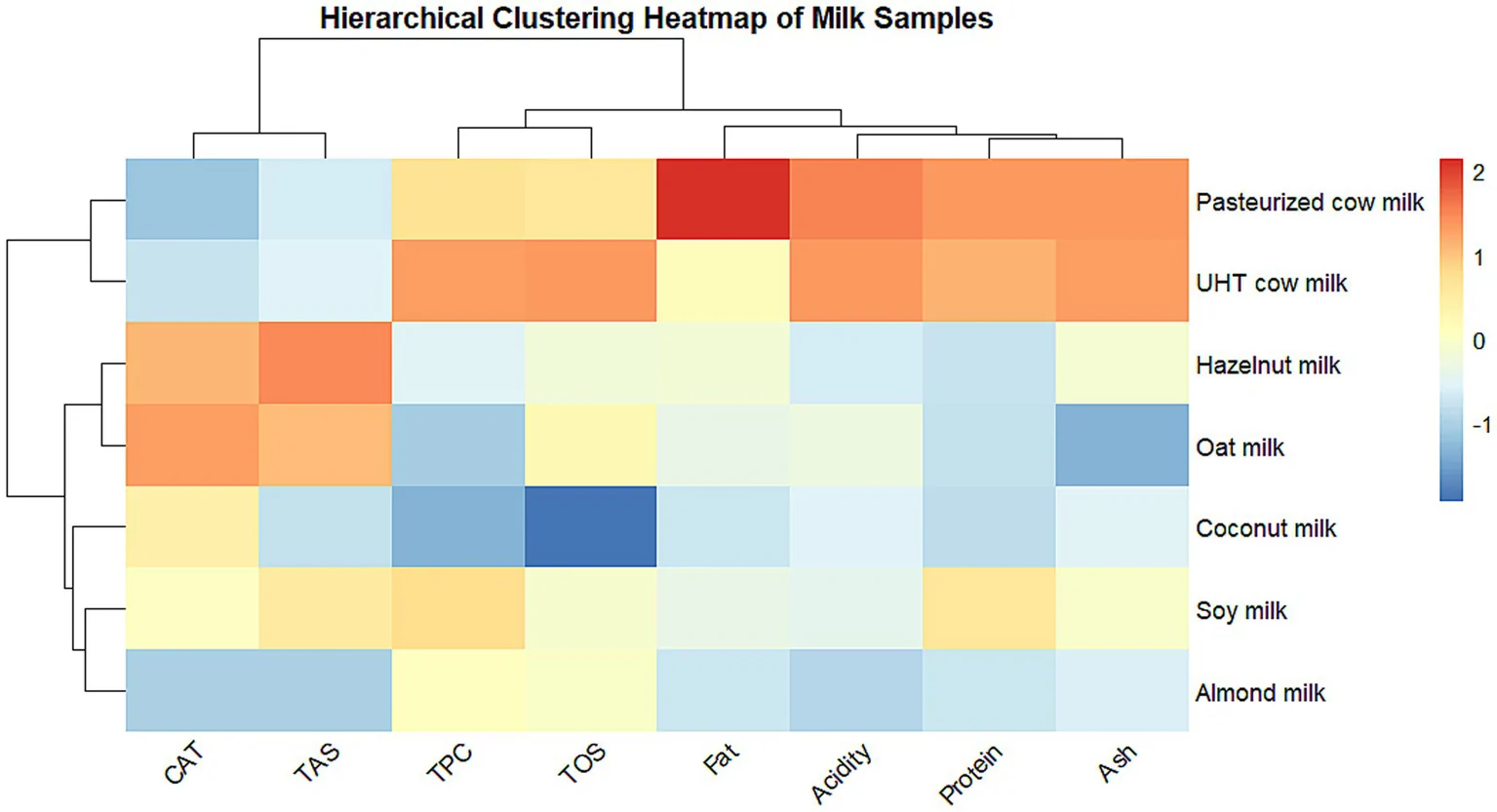
Hierarchical clustering heatmap of physicochemical characteristics, antioxidant capacity, and oxidative status parameters of cow milk and plant-based milk alternatives (variables were standardized using *z*-score transformation prior to clustering. Samples were clustered using Euclidean distance and complete linkage. Red and blue colors indicate relatively higher and lower standardized values, respectively); TPC, total phenolic content; CAT, catalase; TAS, total antioxidant status, TOS, total oxidant status.

### 3.4 Antioxidant–oxidative profiles of individual milk types

The radar plot provided an integrated visualization of the antioxidant and oxidative profiles of the evaluated milk samples (Figure 4). Considerable variability was observed among milk types. Hazelnut milk exhibited the most prominent antioxidant profile, characterized by high normalized TAS and CAT values. Oat milk also demonstrated elevated antioxidant capacity, particularly with respect to TAS. In contrast, coconut milk showed generally lower values across most antioxidant-related parameters.

**Figure 4:**
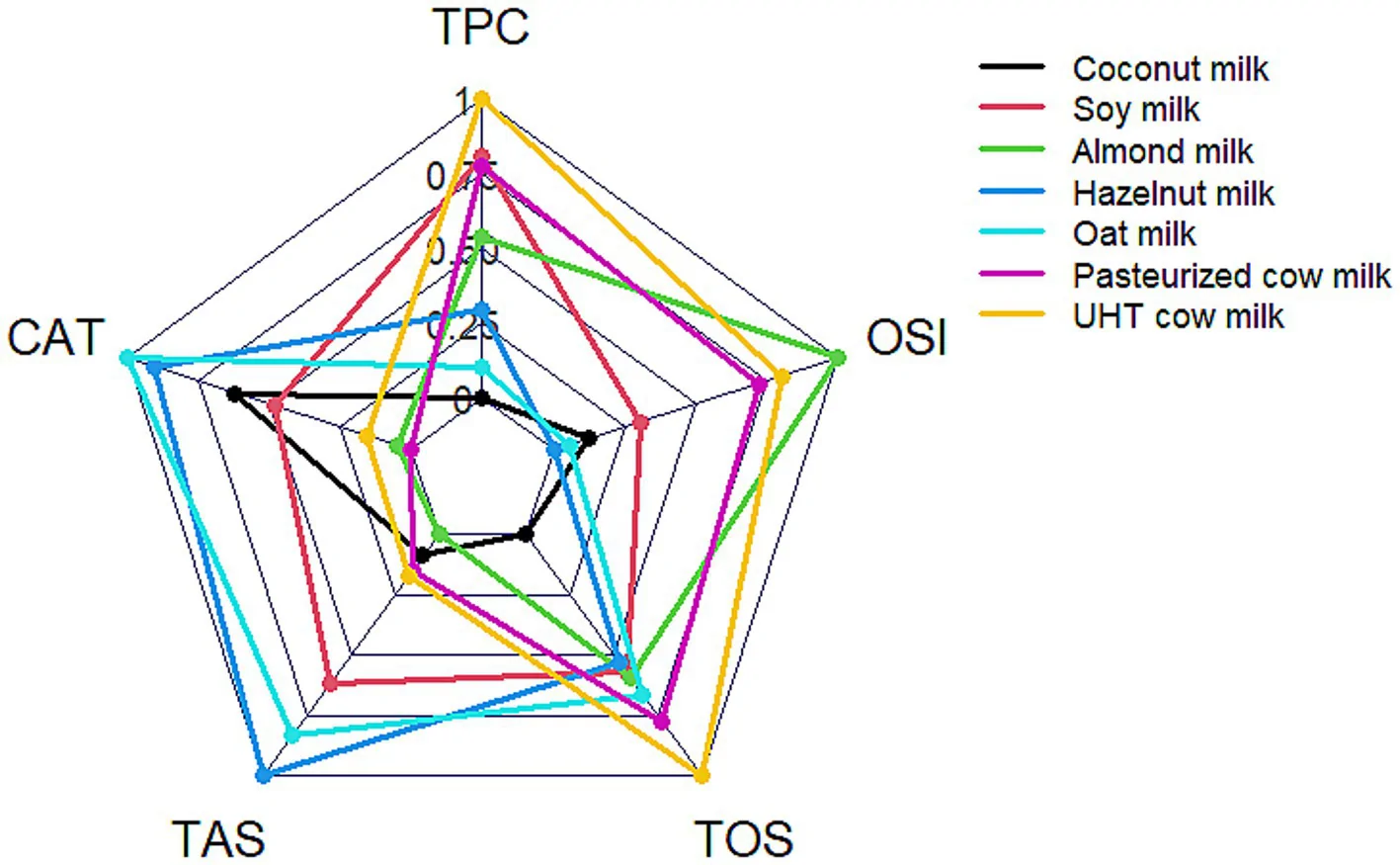
Radar plot showing normalized total phenolic content (TPC), catalase activity (CAT), total antioxidant status (TAS), total oxidant status (TOS), and oxidative stress index (OSI) of cow milk and plant-based milk alternatives.

Cow milk samples, particularly UHT cow milk, displayed higher normalized TOS and OSI values compared with most PBM alternatives, indicating a relatively greater oxidative burden. Soy milk showed an intermediate profile, combining relatively high TPC values with moderate antioxidant and oxidative characteristics. Overall, the radar plot highlighted substantial heterogeneity among PBM alternatives and demonstrated distinct antioxidant–oxidative patterns across milk types.

### 3.5 Comparison between cow milk and plant-based milk alternatives

To further compare dairy and plant-based products, milk samples were grouped as cow milk (pasteurized and UHT milk) and PBM alternatives (coconut, soy, almond, hazelnut, and oat milk) (Table 2). Cow milk exhibited significantly higher acidity, protein, fat, ash, and TPC than PBM alternatives (all *p* < 0.001). In contrast, CAT activity was significantly higher in PBM alternatives than in cow milk (*p* = 0.002). Although TAS did not differ significantly between the two groups, cow milk showed significantly higher TOS and OSI values than PBM alternatives (both *p* < 0.001). These findings indicate that PBM alternatives had comparable TAS values but higher CAT activity, while exhibiting a lower oxidative burden and oxidative stress index than cow milk.

**Table 2:** Comparison of physicochemical characteristics, antioxidant capacity, and oxidative status between cow milk and plant-based milk alternatives.

Variable	Cow milk (*n* = 16)	Plant-based milk alternatives (*n* = 40)	*p*-value
Acidity (% LA)	0.16 (0.15–0.16)	0.09 (0.07–0.10)	<0.001
Protein (%)	3.10 (3.01–3.23)	0.49 (0.38–0.78)	<0.001
Fat (%)	3.02 (2.10–3.41)	1.40 (1.17–1.60)	<0.001
Ash (%)	0.70 (0.69–0.72)	0.45 (0.35–0.52)	<0.001
TPC (mg GAE/100 mL)	15.20 (13.50–17.10)	7.20 (4.05–12.90)	<0.001
CAT (U/L)	36.35 (28.07–38.92)	42.67 (38.03–53.22)	0.002
TAS (mmol TE/L)	0.21 (0.15–0.27)	0.27 (0.14–0.72)	0.162
TOS (μmol H₂O₂ Eq/L)	14.83 (12.95–17.62)	9.19 (4.66–12.93)	<0.001
OSI	7.46 (6.32–9.33)	1.80 (1.40–6.72)	<0.001

Data are presented as median (IQR). Cow milk, *n* = 16; plant-based milk alternatives, *n* = 40. IQR, interquartile range; TPC, total phenolic content; GAE, gallic acid equivalents; CAT, catalase; TAS, total antioxidant status; TE, Trolox equivalent; TOS, total oxidant status; Eq, equivalent; OSI, oxidative stress index.

### 3.6 Determinants of oxidative stress index

A multivariable linear regression model was constructed to investigate factors independently associated with OSI across all milk samples (Table 3). The model explained 51.7% of the variability in OSI (*R*^2^ = 0.517; adjusted *R*^2^ = 0.468; *F* = 10.68, *p* < 0.001). CAT activity emerged as the strongest independent predictor of OSI (standardized *β* = −0.48; unstandardized *β* = −0.141, *p* < 0.001). Higher CAT activity was associated with lower oxidative stress. TPC was positively associated with OSI (standardized *β* = 0.33; unstandardized *β* = 0.194, *p* = 0.023), indicating that samples with higher phenolic content tended to exhibit higher OSI values after adjustment for the other variables.

**Table 3:** Multivariable linear regression analysis of factors associated with oxidative stress index (OSI).

Predictor	Unstandardized *β*	SE	Standardized *β*	95% CI	*p*-value
Intercept	5.729	3.394	–	−1.09 to 12.55	0.098
Protein (%)	1.099	0.974	0.41	−0.86 to 3.06	0.265
TPC (mg GAE/100 mL)	0.194	0.083	0.33	0.03 to 0.36	0.023
CAT (U/L)	−0.141	0.033	−0.48	−0.21 to −0.07	<0.001
Soy milk^†^	−1.258	1.423	−0.36	−4.12 to 1.60	0.381
Other plant-based milks^†^	2.615	2.800	0.75	−3.01 to 8.24	0.355

Model performance statistics: *R*^2^ = 0.517; Adjusted *R*^2^ = 0.468; Residual standard error = 2.539; *F* (5,50) = 10.68; Overall model *p* < 0.001; ^†^Reference category: Cow milk. TPC, total phenolic content; GAE, gallic acid equivalent; CAT, catalase; *β*, regression coefficient; SE, standard error; CI, confidence interval; *R*^2^, coefficient of determination.

Protein content was not independently associated with OSI (*p* = 0.265). Likewise, milk category was not significantly associated with oxidative stress after adjustment. Compared with cow milk, neither soy milk (unstandardized *β* = −1.258, *p* = 0.381) nor other PBM alternatives (unstandardized *β* = 2.615, *p* = 0.355) showed significant independent associations with OSI. Overall, these findings indicate that oxidative stress was more strongly related to antioxidant-related characteristics, particularly CAT activity, than to milk category itself.

### 3.7 Global antioxidant quality index

As an exploratory composite metric developed for this study, higher GAQI values indicate a more favorable antioxidant-oxidative profile. The GAQI values differed significantly among milk types (Kruskal–Wallis *χ*^2^ = 22.46, df = 6, *p* = 0.001) (Figure 5). Hazelnut milk exhibited the highest mean GAQI value (1.62 ± 1.08), followed by soy milk (1.19 ± 2.42) and oat milk (0.70 ± 0.97), indicating a more favorable overall antioxidant–oxidative profile. Coconut milk showed an intermediate score (−0.17 ± 1.12). In contrast, almond milk demonstrated the lowest GAQI value (−1.44 ± 1.23). Both pasteurized cow milk (−1.10 ± 1.56) and UHT cow milk (−0.80 ± 1.28) also exhibited negative GAQI values, reflecting a less favorable antioxidant–oxidative balance. When milk samples were grouped as cow milk and PBM alternatives, plant-based products showed significantly higher GAQI values than cow milk samples (median: 0.58 vs. −1.14, *p* = 0.006). These findings suggest that PBM alternatives generally possess a more favorable antioxidant–oxidative profile than cow milk.

**Figure 5:**
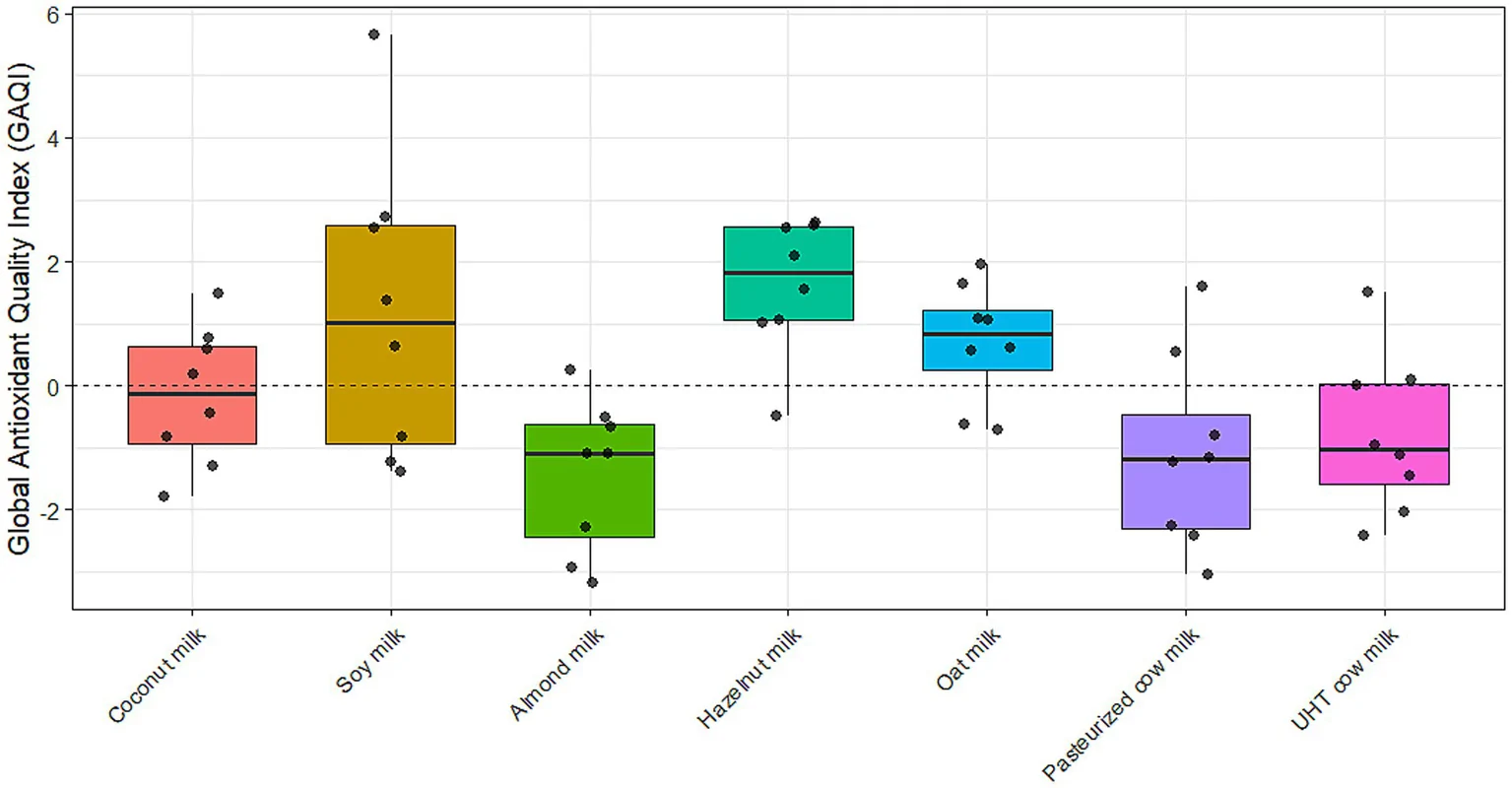
Global Antioxidant Quality Index (GAQI) across cow milk and plant-based milk alternatives. Higher GAQI values indicate a more favorable antioxidant–oxidative profile (GAQI was calculated as (zTPC + zCAT + zTAS) − zTOS. The horizontal dashed line represents a GAQI value of zero).

## 4 Discussion

Milk is widely recognized as a nutrient-rich food because of its balanced composition of water, macronutrients, micronutrients, and bioactive compounds, and it has been associated with nutritional, immunological, and anti-inflammatory properties. In recent years, PBM alternatives have gained increasing popularity; however, their physicochemical and nutritional characteristics differ substantially from those of cow milk because of differences in raw materials and manufacturing processes.

In the present study, titratable acidity differed significantly among milk types, with cow milk exhibiting higher acidity values than all PBMs. This finding agrees with previous reports showing that dairy milk generally has higher titratable acidity than plant-based beverages such as almond and soy milks (5, 30). These differences have been attributed to the relatively higher buffering capacity of cow milk, which is related to its proteins, phosphate–citrate systems, mineral salts, and organic acids such as lactic acid (5). In contrast, PBMs generally have different buffering systems and do not contain lactose, which contributes to differences in titratable acidity (30, 31). PBMs, which primarily contain plant-derived carbohydrates, proteins, and lipids, exhibit different acid–base properties and buffering characteristics from cow milk (5, 31–33). Although previous studies have suggested that soy and coconut milks may exhibit relatively higher titratable acidity than cereal- or nut-based beverages, these values are still significantly lower than those of cow milk (30). However, in the present study, acidity values among PBMs were statistically similar, suggesting that commercial formulation and processing practices may contribute substantially to acidity variation in addition to botanical origin.

Protein content differed markedly among milk types, with median values of 3.25% in pasteurized cow milk, 3.08% in UHT cow milk, and 2.60% in soy milk, compared with 0.28–0.54% in coconut, almond, hazelnut, and oat milks. This pattern is consistent with previous studies demonstrating that soy beverages are distinctive among PBMs because their protein concentration can approach that of cow milk. Soy protein also has a comparatively favorable essential-amino-acid profile for a plant protein, particularly because of its relatively high lysine content; however, sulfur-containing amino acids, especially methionine and cysteine, remain relatively limiting compared with animal proteins (34, 35). Da Silva et al. (7) similarly reported that PBMs generally contain significantly lower protein levels (0.7–1.1 g/100 g) compared to cow milk (2.7–6.2 g/100 g). Jeske et al. (36) reported wide ranges in the macronutrient composition of commercial PBMs, with protein contents ranging from 0.41–2.11% in almond milk, 2.61–3.70% in soy milk, 0.08% in coconut milk, 0.36% in hazelnut milk, and approximately 0.70% in oat milk. In the same study, pasteurized cow milk was reported to contain about 3.70% protein, confirming its substantially higher protein content compared to most plant-based alternatives. These findings are broadly consistent with the protein levels observed in the present study. Similarly, Milovanović et al. (5) reported that soy milk contains higher protein and fat contents compared to other PBMs, while pasteurized cow milk consistently exhibits higher overall macronutrient levels. Lower protein concentrations in nut- and cereal-based milks are largely a function of the raw material composition and formulation practices, as nuts and cereals typically contain more lipids and carbohydrates and less protein than legumes or dairy milk (36, 37). Differences in raw-material composition, extraction conditions, dilution, filtration, and formulation may also contribute to the heterogeneity in protein content among commercial PBMs.

Fat content also differed significantly among milk types (*p* < 0.001). Pasteurized cow milk exhibited the highest median fat content (3.27%), whereas UHT cow milk and most PBMs showed lower values. Differences in fat content and lipid composition among commercial products may arise from several factors, including at standardization, which adjusts the cream-to-skim ratios to achieve a target fat level; homogenization which reduces fat-globule size and redistributes membrane material; PBM formulation, which determines the amount and type of added vegetable oils; and filtration, dilution, and heat treatment, which may affect the recovery and physical state of lipid fractions (30, 38). Therefore, the observed differences between pasteurized and UHT cow milk should not be attributed to heat treatment alone, as product formulation and fat standardization may also contribute. Da Silva et al. (7) reported that PBMs may contain lipid levels comparable to or even higher than those of whole cow milk (2.6 to 4.5 g/100 g vs. ~3.3 g /100 g), depending on raw material selection and product formulation. In the present study, however, PBMs generally exhibited lower fat contents than cow milk, highlighting the heterogeneity of commercially available plant-based beverages. In addition, UHT cow milk showed a wider interquartile range for fat content than pasteurized cow milk (2.92 vs. 0.73 percentage points), indicating greater between-product variability among the UHT products sampled. This greater variability may reflect differences in product formulation and fat standardization among commercial products rather than an effect of UHT processing per se (38, 39).

Ash content, which provides an overall estimate of the mineral residue, was generally higher in cow milk than in most PBMs, particularly oat, almond, and coconut milks. This observation is consistent with the naturally high concentrations of calcium, phosphorus, and associated mineral salts in dairy milk (40). In contrast, PBMs often exhibit lower and more variable mineral contents depending on raw material composition, dilution level, and formulation practices (36, 41). While cow milk samples exhibited relatively narrow interquartile ranges for ash content, PBMs—especially soy milk—showed greater variability. This variability likely reflects differences in raw material composition, mineral fortification strategies, and processing conditions among commercial products. Similar variability patterns in the mineral content of both dairy and PBMs have been reported previously, with cow milk composition being influenced by factors such as breed, feeding regime, and season, whereas plant-based beverages are more strongly affected by formulation and fortification practices (36, 40, 42).

Lower ash values for hazelnut milk have been reported by Aysu et al. (43), who observed ash contents of approximately 0.30%, which are lower than those found in the present study. Such discrepancies can be attributed to differences in formulation, processing, and raw material origin. Supporting this variability, Olagunju and Oyewumi (44) reported ash contents ranging from 0.85% in soy milk to 1.63–2.98% in milk formulations derived from blends of tigernut, cashew nut, and coconut.

Furthermore, Aysu et al. (43) reported wide ranges in physicochemical and antioxidant properties of hazelnut milk, including acidity (0.009–0.054%), protein (2.20–2.45%), fat (2.31–3.54%), ash (0.22–0.30%), TPC (51.45–97.25 mg/L), and antioxidant capacity (55.11–141.97 μmol/L DPPH), further highlighting the strong influence of formulation and processing conditions on the mineral and bioactive composition of PBMs. Milk contains a wide range of antioxidant constituents, including enzymatic antioxidants, phenolic compounds, antioxidant peptides, and vitamins (45). In the present study, cow milk and soy milk exhibited relatively high TPC values compared with oat and coconut milks, supporting previous reports that soy beverages generally contain higher phenolic levels than most nut- and cereal-based alternatives (46, 47). Delgado-Andrade et al. (48) reported that TPC value was in the range of 0.91–1.29 μmol GAE/mL in soy milk samples. Olagunju and Oyewumi (44), who compared soy milk with beverages formulated from various combinations of tigernut, cashewnut, and coconut, also reported that soy milk exhibited higher TPC values than composite beverages; however, among the composite formulations, the blend containing 90% coconut milk showed the highest TPC. In contrast, coconut milk generally shows low phenolic content, which has been attributed to its intrinsic raw material composition and extensive dilution during beverage formulation (13). Nevertheless, substantial differences in TPC among studies likely arise from variation in raw materials, processing conditions, formulation strategies, and analytical methodologies. Vazquez et al. (45) reported TPC values of approximately 49 mg GAE/L in cow milk, with higher values observed in goat and sheep milk, emphasizing the influence of animal species, feeding regime, and phenolic metabolism. Similarly, El-Menawy et al. (49) reported lower TPC values in fermented cow milk compared to fermented quinoa milk, suggesting that both raw material origin and processing conditions strongly affect phenolic retention. In the present study, TPC values of pasteurized and UHT cow milk were lower than those reported by Ertan et al. (50) for UHT skim and pasteurized full-fat cow milk. Similarly, the TPC values observed in the present study were lower than those reported by Sik et al. (42) for raw cow milk. Such differences may reflect variation in milk type, processing conditions, raw material characteristics, and analytical methodology. The presence of phenolic compounds in milk of animal origin and dairy products has been associated with several factors, including the consumption of phenolic-rich fodder crops and dietary incorporation (51–53), ruminal biotransformation of dietary phenolics (54), process-related supplementation, and the intentional addition of phenolic compounds as functional ingredients (55). In PBM alternatives, processing steps such as soaking, blanching, filtration, and enzymatic treatments may contribute to reductions in phenolic content through partial degradation or removal of soluble phenolics, resulting in lower TPC levels compared to raw plant materials (15, 53).

TAS values also differed significantly among milk types. Hazelnut and oat milks exhibited the highest TAS values, whereas almond milk showed the lowest. These findings suggest that antioxidant capacity depends not only on phenolic concentration but also on the combined contribution of other bioactive constituents, including tocopherols and flavonoids, as well as matrix composition and processing effects (56–58). In contrast, cow milk samples, particularly UHT cow milk, exhibited higher TOS, while coconut milk showed the lowest oxidant levels. Consequently, OSI values tended to be higher in cow milk and almond milk than in other PBMs. Although OSI values were similar between pasteurized and UHT milk, the higher TOS observed in UHT milk suggests that product formulation and composition may contribute substantially to oxidative status in addition to processing conditions (50, 59). Ağagündüz (60) reported TAS, TOS, and OSI values of 0.54 mmol TE/L, 14.90 μmol H₂O₂/L, and 2.78, respectively, in full-fat cow milk, while reduced-fat milk showed slightly lower TAS and higher OSI values. Eğritağ et al. (61) reported lower TAS values in cow milk compared to those observed in the present study. In the current work, TOS values for cow milk were comparable to those reported by Ağagündüz (60); however, relatively lower TAS values resulted in higher OSI levels. Overall, comparisons with the literature indicate considerable variability in TAS, TOS, and OSI values, likely arising from differences in analytical methodology, milk composition, animal diet, fat content, and processing conditions (60, 61).

CAT, an important enzymatic antioxidant, is a heme-containing antioxidant enzyme present in both animal- and plant-derived foods and plays a critical role in cellular defense against oxidative damage by catalyzing the decomposition of hydrogen peroxide into water and oxygen (62). CAT differed significantly among milk types. Hazelnut and oat milks demonstrated higher CAT activity than cow milk and almond milk, indicating a stronger measured enzymatic antioxidant potential under the analytical conditions used in this study. Kazak and Coşkun (63) reported the CAT activity of 2.39 U/mL in raw milk from Simmental cows. This value is not directly comparable with the present results because assay methodology, and reporting units may substantially affect CAT activity estimates. Reported CAT activities may also vary considerably across studies because of differences in animal characteristics, processing conditions, sample preparation, and analytical methods (62, 64).

Correlation analysis further revealed that the relationships among physicochemical properties, phenolic content, antioxidant capacity, and oxidative stress parameters varied considerably among milk types, highlighting the complexity of antioxidant regulation in both dairy and plant-based beverages. Delgado-Andrade et al. (48) reported a strong and significant positive correlation between TPC and antioxidant parameters in soy milks, supporting the contribution of phenolic compounds to the overall antioxidant potential of soy-based milks. However, the strength and significance of these relationships varied among milk types, indicating that phenolics alone do not universally explain antioxidant behavior across all matrices. Conversely, negative correlations between TAS and OSI in cow and soy milk were consistent with an inverse association between antioxidant capacity and oxidative stress, in agreement with previous reports in both dairy milk and PBMs (45, 50, 65).

Previous studies have shown that macronutrient composition may influence antioxidant-related parameters. Karlsson et al. (39) reported a strong positive correlation between protein and fat contents in cow milk (*p* < 0.001), reflecting the structural association of milk fat globules with milk proteins. Similarly, Ertan et al. (50) demonstrated strong positive correlations between fat content, antioxidant capacity, and TPC in various milk samples (*p* < 0.0001), suggesting that lipid-associated antioxidants and fat-soluble bioactives may contribute to overall antioxidant potential.

Processing conditions appear to modulate these relationships. Xu and Chang (66) reported that no significant correlations were observed between TPC and antioxidant activity in UHT-processed soy milk, whereas strong linear correlations were present in traditionally processed soy milk. They concluded that UHT processing alters the retention, distribution, and functionality of phenolic compounds and antioxidant activity, highlighting the importance of processing intensity on antioxidant behavior. This observation is consistent with the variability in correlations observed between differently processed milk samples in the present study.

In coconut milk, the strong positive correlation between ash content and OSI suggests that mineral composition may influence oxidative processes. Certain trace minerals can exert pro-oxidant effects by catalyzing oxidative reactions, thereby increasing oxidative stress under specific conditions. Although the present study did not quantify individual minerals, the observed association indicates a potential role of mineral-mediated oxidation in coconut milk systems. In hazelnut milk, strong interrelationships among ash content, TAS, TOS, CAT activity, and TPC highlight the importance of seed structure and compositional density. Hazelnuts are rich in minerals, phenolics, and enzymatic antioxidants, and their concurrent associations suggest that antioxidant capacity in hazelnut milk may reflect the combined influence of multiple compositional factors rather than a single measured component.

Thermal processing is also known to affect antioxidant-related parameters by modifying protein structure and enzyme activity. Ertan et al. (50) reported significant positive correlations between antioxidant activity and TPC in UHT milk, indicating that heat-induced changes in milk constituents may enhance or redistribute antioxidant-active components. Similarly, Alyaqoubi et al. (65) reported strong positive correlations between TPC and antioxidant activity parameters in goat milk, further supporting the contribution of phenolic compounds to antioxidant behavior in dairy systems.

The observed correlation patterns also indicate that antioxidant behavior cannot be explained by phenolic compounds alone. Instead, oxidative balance appears to result from the combined influence of enzymatic antioxidants, mineral-related characteristics, macronutrient composition, phenolic constituents, and processing conditions, underscoring the multifactorial nature of antioxidant regulation in both dairy and PBMs.

The multivariate analyses demonstrated that milk samples were differentiated according to both their compositional characteristics and antioxidant–oxidative properties. PCA identified protein, ash, acidity, and phenolic content as the main contributors to the first principal component, whereas TAS and CAT activity contributed predominantly to the second component, indicating that antioxidant-related variables contributed substantially to a distinct dimension of variation among milk types. These findings are consistent with previous studies showing that multivariate statistical approaches effectively distinguish dairy milk from plant-based beverages and reveal compositional heterogeneity among different PBM categories (67, 68). The close clustering of pasteurized and UHT cow milk in both the PCA and hierarchical clustering analyses suggests that differences between dairy and plant-based products were more prominent than those observed between the two cow milk processing types. This finding is consistent with previous studies showing that multivariate analytical approaches can effectively discriminate dairy milk from plant-based beverages based on their compositional characteristics (69). In contrast, hazelnut and oat milks clustered together and were associated with antioxidant-related variables, whereas coconut and almond milks were characterized by lower protein, mineral, and antioxidant-related parameters. Soy milk occupied an intermediate position between cow milk and the remaining plant-based alternatives, reflecting its relatively high protein content while maintaining a distinct antioxidant-oxidative profile. Collectively, these findings demonstrate that PBMs constitute a heterogeneous group and should not be evaluated as nutritionally equivalent products, as their compositional and functional characteristics vary considerably according to botanical source and formulation strategies (36).

The radar plot complemented the multivariate analyses by providing an integrated visualization of antioxidant and oxidative characteristics. Hazelnut and oat milks displayed more favorable antioxidant profiles, primarily because of their higher TAS and CAT values, whereas coconut milk exhibited comparatively lower antioxidant-related measurements. Cow milk samples showed relatively higher TOS and OSI values, indicating a greater measured oxidative burden despite their favorable nutritional composition. Soy milk again occupied an intermediate position in the multivariate analyses, reflecting its relatively high protein and phenolic contents alongside a distinct combination of antioxidant and oxidative characteristics.

Comparison of grouped data further highlighted the contrast between nutritional composition and oxidative characteristics. Cow milk generally exhibited higher protein, fat, mineral (ash), and phenolic contents than most PBMs. Nevertheless, it also showed significantly higher oxidant status and oxidative stress indices. By comparison, PBMs displayed higher CAT activity and lower TOS and OSI values, while maintaining TAS values that were broadly comparable to those of cow milk. These observations suggest that conventional nutritional composition alone may not fully reflect the oxidative characteristics of milk products.

The multivariable regression analysis provided additional insight into factors independently associated with OSI. After adjustment for compositional and antioxidant-related variables, milk category was not independently associated with OSI, indicating that the observed differences in oxidative status may be more closely related to measured compositional characteristics than to the origin of the beverage itself. Among the evaluated predictors, CAT activity emerged as the strongest independent predictor of OSI, exhibiting a significant inverse association with OSI, whereas TPC showed a more modest positive association. These findings emphasize the importance of enzymatic antioxidant activity in the observed oxidative balance, consistent with previous reports identifying CAT as an important contributor to the antioxidant defense capacity of milk (62, 64), and support the results obtained from the correlation and multivariate analyses.

The composite GAQI offered an integrated assessment of antioxidant quality by simultaneously incorporating phenolic content, CAT activity, TAS, and oxidative burden into a single metric. Hazelnut milk achieved the highest GAQI score, followed by soy and oat milk, whereas almond milk and both cow milk groups showed comparatively lower scores. The broadly consistent patterns observed across the GAQI, PCA, hierarchical clustering, and radar plot analyses provide convergent descriptive evidence of differences in antioxidant-oxidative profiles among milk types. However, because GAQI was developed specifically for this study as an exploratory composite index, further validation using independent datasets and broader product categories is warranted before its broader application.

### 4.1 Study limitations

This study was designed to compare physicochemical and antioxidant–oxidative characteristics and therefore did not evaluate organoleptic properties, consumer acceptance or purchasing preferences, microbiological quality, or rheological behavior. In addition, the sample size was limited to eight commercial products per milk type, and products were obtained from the Turkish market, which may limit generalizability to other brands, formulations, production batches, and countries. Processing conditions should also be considered when interpreting the observed differences among milk categories. All PBMs evaluated in the present study were commercially UHT-treated products, whereas cow milk included both pasteurized and UHT-treated samples. Heat treatment may influence protein structure, phenolic compounds, endogenous antioxidant systems, and oxidation-related parameters and may therefore have contributed to some of the observed differences. However, differences were also observed between UHT cow milk and UHT-treated PBMs, suggesting that processing category alone does not account for the observed patterns and that differences in raw-material composition, formulation, and other product-specific processing steps may also contribute. Because detailed industrial processing parameters, including exact time–temperature combinations, were not available, the independent contribution of processing intensity could not be determined. Manufacturer-specific formulation and processing conditions were not experimentally controlled, and the cross-sectional laboratory design does not permit inference regarding health effects in consumers. Finally, GAQI is an exploratory, study-specific composite index and requires external validation before broader use.

## 5 Conclusion

In conclusion, significant differences were observed among dairy milks and PBMs in terms of physicochemical properties, antioxidant capacity, and oxidative stress parameters. Cow milk and soy milk exhibited the highest protein contents, whereas nut- and cereal-based milks contained substantially lower protein levels. Pasteurized cow milk showed the highest fat content, while most PBMs had lower and relatively similar fat values. Hazelnut and oat milks exhibited the highest TAS values despite relatively modest TPC levels, suggesting that factors beyond TPC may contribute to overall antioxidant capacity. In contrast, UHT cow milk showed the highest TOS, whereas coconut milk exhibited the lowest TOS. Cow milk also showed relatively high OSI values, while coconut and most PBMs had lower OSI values. Correlation and multivariate analyses demonstrated that antioxidant capacity and oxidative balance are associated with multiple compositional factors, including phenolic content, enzymatic antioxidant activity, and ash-related compositional characteristics, and that these relationships differ among milk types. Overall, within the analytical scope of this study, the findings highlight the marked heterogeneity of PBMs and emphasize the importance of evaluating both nutritional composition and antioxidant-oxidative properties when comparing milk alternatives.

## Data Availability

The raw data supporting the conclusions of this article will be made available by the authors, without undue reservation.
