# Supplementary material for: Physicochemical characteristics, antioxidant capacity, and oxidative stress profiles of dairy and plant-based milks: a comparative multivariate analysis
Source: Front Nutr. 2026 Oct 23;13:1948308. doi: 10.3389/fnut.2026.1948308 (PMC13739664; doi:10.3389/fnut.2026.1948308)
Supplement: Supplementary file 1 [file Supplementary_file_1.docx]

**Supplementary Figure 1.**

Sample-level hierarchical clustering heatmap of physicochemical characteristics, antioxidant capacity, and oxidative status parameters across all milk samples (n = 56). Variables were standardized using z-score transformation prior to clustering. Red and blue colors indicate relatively higher and lower values, respectively.
