# Supplementary material for: Physicochemical characteristics, antioxidant capacity, and oxidative stress profiles of dairy and plant-based milks: a comparative multivariate analysis
Source: Front Nutr. 2026 Oct 23;13:1948308. doi: 10.3389/fnut.2026.1948308 (PMC13739664; doi:10.3389/fnut.2026.1948308)
Supplement: Supplementary file 2 [file Table_1.docx]

Supplementary Table 1. Milk type-specific Spearman correlation coefficients among physicochemical characteristics, antioxidant capacity, and oxidative stress parameters.

| Milk Type | Protein | Fat | Ash | TPC | CAT | TAS | TOS | OSI |
| --- | --- | --- | --- | --- | --- | --- | --- | --- |
| Coconut milk | |  |  |  |  |  |  |  |
| Acidity | 0.73* | 0.67 | -0.54 | 0.92** | 0.13 | 0.83* | 0.07 | -0.58 |
| Protein |  | 0.62 | -0.33 | 0.73* | 0.10 | 0.76* | 0.29 | -0.33 |
| Fat |  |  | -0.76* | 0.72* | 0.55 | 0.64 | -0.10 | -0.57 |
| Ash |  |  |  | -0.52 | -0.33 | -0.52 | 0.62 | 0.91** |
| TPC |  |  |  |  | 0.17 | 0.90** | 0.13 | -0.58 |
| CAT |  |  |  |  |  | 0.36 | 0.05 | -0.29 |
| TAS |  |  |  |  |  |  | 0.07 | -0.69 |
| TOS |  |  |  |  |  |  |  | 0.60 |
| Soy milk | |  |  |  |  |  |  |  |
| Acidity | 0.00 | 0.17 | 0.42 | -0.36 | -0.22 | -0.18 | 0.08 | 0.43 |
| Protein |  | 0.12 | 0.31 | 0.81* | 0.26 | 0.07 | 0.41 | 0.29 |
| Fat |  |  | 0.05 | 0.07 | 0.43 | 0.24 | 0.17 | 0.17 |
| Ash |  |  |  | 0.26 | -0.14 | -0.48 | 0.00 | 0.48 |
| TPC |  |  |  |  | 0.41 | 0.17 | 0.50 | 0.19 |
| CAT |  |  |  |  |  | 0.76* | -0.12 | -0.41 |
| TAS |  |  |  |  |  |  | -0.21 | -0.71* |
| TOS |  |  |  |  |  |  |  | 0.74* |
| Almond milk | |  |  |  |  |  |  |  |
| Acidity | -0.27 | -0.68 | 0.64 | 0.13 | 0.60 | 0.23 | 0.44 | 0.79* |
| Protein |  | 0.13 | -0.44 | -0.21 | -0.41 | 0.37 | 0.30 | -0.30 |
| Fat |  |  | -0.34 | -0.02 | -0.40 | -0.60 | -0.71 | -0.43 |
| Ash |  |  |  | -0.38 | 0.76* | -0.14 | -0.02 | 0.24 |
| TPC |  |  |  |  | -0.25 | 0.07 | 0.23 | 0.67 |
| CAT |  |  |  |  |  | 0.14 | 0.33 | 0.29 |
| TAS |  |  |  |  |  |  | 0.91** | 0.05 |
| TOS |  |  |  |  |  |  |  | 0.36 |
| Hazelnut milk | |  |  |  |  |  |  |  |
| Acidity | 0.17 | 0.18 | -0.48 | 0.30 | -0.17 | -0.17 | 0.30 | 0.77* |
| Protein |  | 0.27 | 0.01 | 0.17 | 0.14 | 0.14 | 0.17 | 0.46 |
| Fat |  |  | 0.12 | 0.29 | 0.37 | 0.37 | 0.29 | 0.51 |
| Ash |  |  |  | 0.43 | 0.88** | 0.88** | 0.43 | -0.41 |
| TPC |  |  |  |  | .762* | 0.76* | 1.00** | 0.52 |
| CAT |  |  |  |  |  | 1.00** | 0.76* | 0.05 |
| TAS |  |  |  |  |  |  |  | 0.05 |
| TOS |  |  |  |  |  |  |  | 0.52 |
| Oat milk | |  |  |  |  |  |  |  |
| Acidity | 0.08 | -0.09 | -0.87** | -0.70 | 0.36 | -0.41 | -0.49 | 0.01 |
| Protein |  | -0.91** | 0.12 | 0.31 | -0.12 | -0.02 | 0.00 | 0.05 |
| Fat |  |  | -0.12 | -0.33 | 0.38 | 0.21 | 0.12 | -0.19 |
| Ash |  |  |  | 0.95** | -0.55 | 0.24 | 0.41 | 0.05 |
| TPC |  |  |  |  | -0.62 | 0.14 | 0.36 | 0.17 |
| CAT |  |  |  |  |  | 0.41 | 0.17 | -0.21 |
| TAS |  |  |  |  |  |  | 0.95** | 0.48 |
| TOS |  |  |  |  |  |  |  | 0.64 |
| Pasteurized cow milk | | |  |  |  |  |  |  |
| Acidity | 0.30 | 0.11 | 0.46 | -0.63 | 0.44 | 0.44 | 0.55 | -0.03 |
| Protein |  | -0.05 | 0.69 | -0.02 | 0.31 | 0.10 | 0.19 | 0.24 |
| Fat |  |  | 0.29 | 0.17 | 0.14 | -0.79* | -0.14 | 0.55 |
| Ash |  |  |  | 0.14 | 0.14 | -0.17 | 0.29 | 0.48 |
| TPC |  |  |  |  | -0.43 | -0.52 | -0.60 | 0.02 |
| CAT |  |  |  |  |  | 0.29 | -0.29 | -0.26 |
| TAS |  |  |  |  |  |  | 0.21 | -0.71* |
| TOS |  |  |  |  |  |  |  | 0.45 |
| UHT cow milk | |  |  |  |  |  |  |  |
| Acidity | -0.47 | 0.22 | -0.25 | -0.63 | -0.50 | 0.23 | 0.06 | -0.43 |
| Protein |  | 0.35 | -0.58 | -0.08 | 0.24 | 0.69 | 0.77* | -0.41 |
| Fat |  |  | -0.57 | -0.67 | -0.02 | 0.37 | 0.19 | -0.52 |
| Ash |  |  |  | 0.43 | -0.29 | -0.64 | -0.74* | 0.57 |
| TPC |  |  |  |  | 0.50 | -0.45 | -0.31 | 0.50 |
| CAT |  |  |  |  |  | -0.13 | 0.00 | 0.05 |
| TAS |  |  |  |  |  |  | 0.88** | -0.88** |
| TOS |  |  |  |  |  |  |  | -0.60 |

Values represent Spearman's rank correlation coefficients (ρ). Each milk type includes eight samples (n = 8). *p < 0.05; **p < 0.01. TPC, total phenolic content; CAT, catalase activity; TAS, total antioxidant status; TOS, total oxidant status; OSI, oxidative stress index.
