# Supplementary material for: Physicochemical characteristics, antioxidant capacity, and oxidative stress profiles of dairy and plant-based milks: a comparative multivariate analysis
Source: Front Nutr. 2026 Oct 23;13:1948308. doi: 10.3389/fnut.2026.1948308 (PMC13739664; doi:10.3389/fnut.2026.1948308)
Supplement: Supplementary file 3 [file Table_2.docx]

**Supplementary Table 2.** Variable Loadings and relative contributions of physicochemical, antioxidant, and oxidative parameters to PC1 and PC2.

| Variable | PC1 Loading | PC1 Contribution  (%) | PC2 Loading | PC2 Contribution  (%) |
| --- | --- | --- | --- | --- |
| Acidity | 0.755 | 15.6 | 0.039 | 0.1 |
| Protein | 0.892 | 21.8 | 0.138 | 1.2 |
| Fat | 0.593 | 9.6 | 0.251 | 4.0 |
| Ash | 0.827 | 18.7 | 0.006 | 0.0 |
| TPC | 0.741 | 15.0 | 0.091 | 0.5 |
| CAT | -0.553 | 8.4 | 0.676 | 29.0 |
| TAS | -0.331 | 3.0 | 0.863 | 47.1 |
| TOS | 0.535 | 7.8 | 0.534 | 18.1 |

TPC: total phenolic content; CAT: catalase; TAS: total antioxidant status; TOS: total oxidant status; PC1: first principal component; PC2: second principal component
